# Hsp40s Specify Functions of Hsp104 and Hsp90 Protein Chaperone Machines

**DOI:** 10.1371/journal.pgen.1004720

**Published:** 2014-10-16

**Authors:** Michael Reidy, Ruchika Sharma, Shankar Shastry, Brittany-Lee Roberts, Ivan Albino-Flores, Sue Wickner, Daniel C. Masison

**Affiliations:** 1 Laboratory of Biochemistry and Genetics, National Institute of Diabetes and Digestive and Kidney Diseases, Bethesda, Maryland, United States of America; 2 Laboratory of Molecular Biology, National Cancer Institute, National Institutes of Health, Bethesda, Maryland, United States of America; The University of Arizona, United States of America

## Abstract

Hsp100 family chaperones of microorganisms and plants cooperate with the Hsp70/Hsp40/NEF system to resolubilize and reactivate stress-denatured proteins. In yeast this machinery also promotes propagation of prions by fragmenting prion polymers. We previously showed the bacterial Hsp100 machinery cooperates with the yeast Hsp40 Ydj1 to support yeast thermotolerance and with the yeast Hsp40 Sis1 to propagate [*PSI^+^*] prions. Here we find these Hsp40s similarly directed specific activities of the yeast Hsp104-based machinery. By assessing the ability of Ydj1-Sis1 hybrid proteins to complement Ydj1 and Sis1 functions we show their C-terminal substrate-binding domains determined distinctions in these and other cellular functions of Ydj1 and Sis1. We find propagation of [URE3] prions was acutely sensitive to alterations in Sis1 activity, while that of [*PIN^+^*] prions was less sensitive than [URE3], but more sensitive than [*PSI^+^*]. These findings support the ideas that overexpressing Ydj1 cures [URE3] by competing with Sis1 for interaction with the Hsp104-based disaggregation machine, and that different prions rely differently on activity of this machinery, which can explain the various ways they respond to alterations in chaperone function.

## Author Summary

The cellular chaperone machinery helps proteins adopt and maintain native conformations and protects cells from stress. The yeast Hsp40s Ydj1 and Sis1 are co-chaperones that regulate Hsp70s, which are key components of many chaperone complexes. Both of these Hsp40s are crucial for growth and Ydj1 directs disaggregation activity of the Hsp100-based machinery to provide stress protection while Sis1 directs this activity to promote prion replication. Ydj1 also cures yeast of certain prions when overexpressed. We show that C-terminal domains that possess substrate-binding function of Ydj1 and Sis1 can mediate these and other functional distinctions and that the degree that prions depend on Sis1 activities could underlie differences in how they respond to alterations of chaperones. These findings support a view that Hsp40s regulate and specify functions of the chaperone machinery through substrate discrimination and cooperation with Hsp70. The disproportionate evolutionary expansion of Hsp40s (J-proteins) relative to their Hsp70 partners led to a proposal that this amplification allows increased regulation and fine-tuning of chaperone machines for increasingly complex processes. Our findings support this idea and provide insight into fundamental aspects of this cooperation.

## Introduction

The protein disaggregation machinery of microorganisms and plants is driven by an Hsp100-family chaperone that cooperates with Hsp70 and its nucleotide exchange factor (NEF) and J- protein (Hsp40) co-chaperones [1]. This machinery promotes cell survival after environmental stresses that cause proteins to aggregate by extracting proteins individually from aggregates [2]–[4]. Organisms encode multiple Hsp70s, Hsp40s and NEFs and there is much to learn about how these chaperones cooperate and regulate each other's activity to effect protein remodeling and reactivation, and how different combinations of chaperones and co-chaperones determine efficiency and specificity of the machinery.

In yeast, this Hsp104-based resolubilization process also targets prions, which are cellular proteins that propagate as highly structured fibrous protein aggregates called amyloid [5]–[7]. The widely studied prions [URE3], [*PSI^+^*] and [*PIN^+^*] (also known as [*RNQ1^+^*]) are composed of the proteins Ure2, Sup35, and Rnq1, respectively [8]–[10]. Propagation of these and other amyloid-based yeast prions requires proper functioning of the disaggregation machinery [5], [11]–[15], which promotes prion replication by fragmenting fibers into more numerous pieces, or seeds, that continue to propagate the prion state [4], [16], [17].

Hsp70s act in various cellular chaperone machines by binding and releasing hydrophobic surfaces on partially folded proteins. This activity is necessary for essential cellular processes where proteins are partially folded, such as transport across membranes, and for preventing protein aggregation under conditions of stress [18], [19]. Effective interactions of Hsp70 with substrates rely on its regulation by J-proteins and NEFs (reviewed in [20]). The major yeast cytosolic Hsp40s Sis1 and Ydj1 are structurally related J-proteins that function as dimers [21]. Both have an amino-terminal J domain that mediates physical interaction with Hsp70s and an adjacent glycine-phenylalanine (GF) rich region that confers some functional distinction [22], [23]. Both also have carboxy-terminal regions that bind substrates with a specificity that overlaps Hsp70 [24]–[26]. The class I J-protein Ydj1 has a zinc-finger element within its C-terminal region and a CAAX motif at its extreme C-terminus that directs its farnesylation. This modification localizes much of Ydj1 to membranes and influences cooperation of Ydj1 with Hsp90, another abundant and highly conserved protein chaperone [27], [28]. The class II J-protein Sis1 lacks these elements, but has a glycine-methionine (GM) rich extension of its GF domain.

Altering function or abundance of Sis1 or Ydj1 inhibits propagation of prions, but in different ways. By an undefined mechanism, overexpressing Ydj1 causes cells to lose [URE3] and some variants of [*PIN^+^*], but not [*PSI^+^*] [12], [29]. Increasing expression of Sis1 does not destabilize these prions [30], [31]. Depleting Sis1 causes [URE3] and [*PIN^+^*] to be lost rapidly as cells divide, but causes [*PSI^+^*] to be lost gradually and only after a long delay [14]. Additionally, all non-essential functions of Sis1 are dispensable for [*PSI^+^*] propagation, but deleting only the GF region of Sis1 causes cells to lose [*PIN^+^*] [30], [32]. Thus, the way these Hsp40s influence prion propagation goes beyond their general roles of regulating Hsp70. Additionally, when cooperating with *E. coli* disaggregation machinery in yeast, Sis1 is specifically required for prion propagation and Ydj1 for protecting cells from exposure to lethal heat (thermotolerance) [15]. Both Hsp40s are critical for survival and while no other J-protein can compensate for loss of Sis1, Sis1 and other J-proteins, as well as J-domains alone, can improve growth of cells lacking Ydj1 [14], [27], [33], [34]. What defines these functional differences of Sis1 and Ydj1 is uncertain.

Here, we used Sis1-Ydj1 hybrid proteins to identify structural elements that determine the distinct functions of Sis1 and Ydj1 in various cellular processes and systematically assessed the importance of Sis1 activities for propagation of [URE3] and [*PIN^+^*]. We found that the C-terminal regions of Sis1 and Ydj1 possessed functional distinctions that directed the action of the Hsp104 machinery in prion propagation and thermotolerance, and the Hsp90 machinery in galactose-induced transcription. We also found that [URE3] was highly sensitive to alterations of Sis1 and that [*PIN^+^*] was less dependent on Sis1 than [URE3], but more dependent than [*PSI^+^*]. Our results support the idea that differences in ways prions respond to various chaperone alterations can be due to differences in their dependence on the disaggregation machinery.

## Results

### The CTD of Sis1 specifies cooperation with *E. coli* chaperones to propagate prions

Earlier we showed that the *E. coli* disaggregation machinery (ClpB, DnaK and GrpE, which are analogous to yeast Hsp104, Hsp70, and NEF, respectively) function in yeast by cooperating with yeast Hsp40s [15]. ClpB, DnaK and GrpE are herein abbreviated B, K and E, respectively. We modified this system to use a DnaK mutant (R167H, designated K*) that can interact only with J-proteins that have the compensatory D36N J-domain mutation (designated Sis1* and Ydj1*). BK*E cannot cooperate with wild type J-proteins, so we can monitor interactions of the BK*E machinery specifically with Sis1* or Ydj1* even in the presence of their wild type counterparts. Using this system we showed BK*E cooperates specifically with Sis1* to propagate [*PSI^+^*] prions and with Ydj1* to protect cells from exposure to lethal heat (thermotolerance). The ability of Sis1 and Ydj1 to direct activities of the disaggregation machinery could be due to differences in the ways they recruit or regulate Hsp70 components of the machinery or interact with different types of substrates. To determine the basis of these and other functional differences we used Sis1-Ydj1 hybrid proteins.

**Figure 1:**
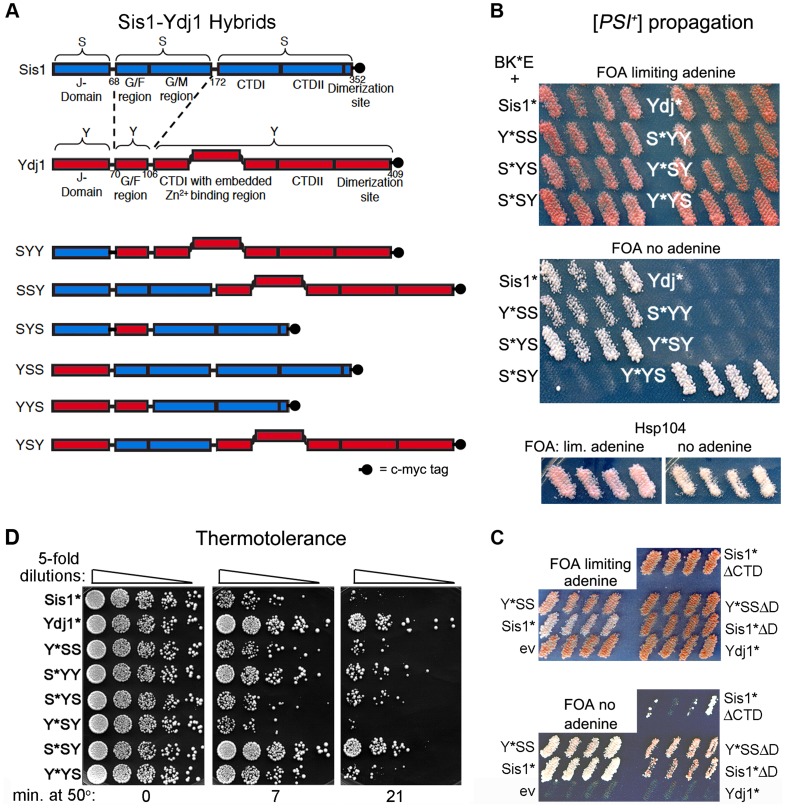
Sis1/Ydj1 hybrid proteins cooperate with *E. coli* chaperones in distinct processes. (A) Diagram of conserved Hsp40 domains swapped in the hybrid proteins (not to scale). Numbers indicate amino acid positions, dashed lines indicate splice joints. The regions from residues 172–352 of Sis1 and from 106–409 of Ydj1 are referred to inclusively as CTD. All alleles contain C-terminal c-myc tags (indicated as filled black circles). (B) [*PSI^+^*] propagation: [*PSI^+^*] *hsp104Δ* strain MR386 expressing Hsp104 on a *URA3* plasmid and the *E. coli* chaperones ClpB, DnaK* and GrpE (BK*E) was transformed by single-copy *TRP1* plasmids expressing D36N versions (*) of indicated wild type and hybrid proteins. For each hybrid, four independent transformants were grown as patches on plates containing uracil to allow loss of the plasmid encoding Hsp104. These were replica-plated onto medium containing FOA with (upper panel) or lacking (lower panel) adenine. Only [*PSI^+^*] cells grow on medium lacking adenine. As reported earlier [15], the Sis1* and Ydj1* cells show representative [*PSI^+^*] and [*psi^−^*] phenotypes, respectively, with ClpB in place of Hsp104. Control cells with Hsp104 on the *TRP1* plasmid are shown below. (C) Transformants expressing Sis1* lacking its CTD (Sis1*ΔCTD) and versions of Sis1*and Y*SS lacking the dimerization domain (ΔD) were processed as in panel (B). (D) Thermotolerance: Transformants from FOA plates in upper image of panel (B) were cured of prions by growth on guanidine-containing medium and then grown in liquid medium selecting for the plasmids. Cells were diluted to similar density, heat shocked at 50°C for the times indicated at the bottom, and five microliters of five-fold serial dilutions were dropped onto YPAD plates and incubated for three days at 30°C.

In earlier work using hybrids of Ydj1 and Sis1 each of the CTDs was divided into two parts (CTDI and CTDII) and the adjacent GM region of Sis1 was included on the same fragment containing the amino-terminal portion of the Sis1 CTD [35], [36]. Exchanging this fragment splits the contiguous functionally redundant GF-GM regions of Sis1 [22], which complicates interpretations of swapping GF domains. In order to simplify comparisons we exchanged only the three most conserved J, GF(GM), and CTD regions to form six hybrid proteins (see Figure 1A, Materials and methods). Hybrids are named according to their structural components. For example, YYS and SSY have their CTDs swapped. All of our J-protein hybrids for the BK*E experiments contain the D36N mutation, which is indicated in their names by an asterisk (e.g. Y*YS is the D36N mutant of YYS).

To test for ability of these modified Sis1-Ydj1 hybrid proteins to cooperate with the *E. coli* chaperones to propagate [*PSI^+^*], they were first expressed in [*PSI^+^*] cells that have ClpB in place of chromosomal Hsp104 and express K*, E and Hsp104 from plasmids. They were then assessed for ability to continue propagating [*PSI^+^*] after the cells lose the plasmid encoding Hsp104. The results are presented in Figure 1B. The upper panel shows cells on medium that allows growth of all strains and the lower panel shows medium that allows growth only of cells propagating [*PSI^+^*]. The lack of distinction among strains in the upper panel indicates that in the absence of selection [*PSI^+^*] propagates too weakly to confer an obvious phenotype, as reported earlier [15]. Nevertheless, the strong confluent growth of cells transferred onto medium selecting for [*PSI^+^*] indicates the prions in these cells are mitotically stable. The presence of [*PSI^+^*] was confirmed by its dominant phenotype in crosses and by guanidine curability (see Figure S1A). Wild type Sis1 and all hybrids containing the CTD of Sis1 propagated [*PSI^+^*]. In contrast, Ydj1 and all proteins with the CTD of Ydj1 were unable to propagate [*PSI^+^*]. Thus, the Sis1-specific function that is necessary for these full-length Hsp40s to cooperate with the bacterial ClpB disaggregation machinery to propagate [*PSI^+^*] prions resides in the Sis1 CTD.

These results were somewhat unexpected because Sis1 lacking its CTD (Sis1ΔCTD) propagates [*PSI^+^*] in cells expressing Hsp104 [32]. We therefore tested if BK*E could cooperate with the truncated Sis1*ΔCTD to propagate [*PSI^+^*]. We found it did, but [*PSI^+^*] was very unstable and was lost rapidly when selection for the prion was not maintained (Figure 1C). These results indicate that Hsp40 requirements of BK*E for [*PSI^+^*] propagation differ when full-length and truncated Hsp40s are used. Inconsistencies between truncated and full-length Hsp40s have been seen before (see below) [22], [30], [33].

Because the dimerization domain is at the C-terminus, it was also possible that Y*YS was able to propagate [*PSI^+^*] only by combining with the wild type Sis1 present in the cells to form heterodimers that functioned with BK*E. Since Sis1 lacking its dimerization domain (Sis1ΔD) propagates [*PSI^+^*] in cells with Hsp104 [32], we tested if the D36N version of Sis1ΔD was able to support [*PSI^+^*] propagation with BK*E. At the same time we tested a version of Y*YS lacking its dimerization domain. Both of these proteins were able to cooperate with BK*E to propagate [*PSI^+^*], but with noticeably reduced efficiency compared with their full-length counterparts (Figure 1C, note slower growth and pink color on medium selecting for [*PSI^+^*]). Thus, Y*YS did not need to dimerize with wild type Sis1 to support propagation of [*PSI^+^*].

### The CTD of Ydj1 allows cooperation with *E. coli* chaperones to provide thermotolerance

Using our hybrids to assess Ydj1 function in thermotolerance we found that, except for Y*SY, proteins containing the CTD of Ydj1 worked better at restoring thermotolerance to *hsp104Δ* cells expressing BK*E (Figure 1D). Overall, the range of survival conferred by the different hybrids was somewhat broad, which suggests that complex interactions among different parts of the proteins can influence Hsp40 functions in this process. Notably, however, S*SY, in which only the CTD is from Ydj1, functioned most like Ydj1 in this assay, while Y*YS performed only slightly better than Sis1*. Thus, as with prion propagation, a determinant of functional specificity between Sis1 and Ydj1 that directs BK*E activity in thermotolerance resides in the CTD.

### The CTD of Sis1 confers Sis1-specific functions in yeast

Although Sis1 is essential for viability, its J, GF and CTD regions are each dispensable for growth of *sis1Δ* cells [22], [30]. However, other work shows that a full-length Sis1-Ydj1 hybrid that contains the substrate-binding region of Sis1, but not Ydj1, supports growth of *sis1Δ* cells [35]. These earlier findings show inconsistencies in the way that Sis1 and Ydj1 sub-regions determine specific Hsp40 functions. The variable dependency of [*PSI^+^*] on the CTD of Sis1 in our full-length versus truncated [32] proteins is another example of such differences. An obvious distinction in these experiments is whether the structural regions are deleted or swapped. While deleting regions allows identifying redundant functions, swapping domains of full-length proteins also allows identifying functions that can be influenced by inter-domain interactions, or if the domains specify interactions with other factors or localization of the J-proteins in the cell.

In line with the earlier findings using full-length hybrid proteins, our results with the *E. coli* chaperones show the CTDs can impart specific functionality to these Hsp40s. To assess which regions are involved in determining specificity of cooperation with the yeast chaperone machinery we tested ability of our hybrids to function in place of Sis1 or Ydj1, which allows investigation of Hsp40 functions other than those needed for prion propagation and thermotolerance.

We compared the relative abundance of the hybrid proteins using western blot analysis (Figure S2). Sis1 and hybrid proteins containing the CTD of Sis1 were less abundant than the others, so we cannot rule out that differences in abundance were contributing to effects in some assays. In addressing this issue by increasing expression using the stronger GPD promoter on both single and high-copy plasmids, we found that hybrid proteins containing the CTD of Sis1 caused growth inhibition of *ydj1Δ* cells in a dose-dependent manner (see Figure S3). Therefore, in our complementation assays we expressed proteins regulated by the *SIS1* promoter on single-copy plasmids. Despite differences in protein levels, in several experiments the less abundant proteins (i.e. those with CTD of Sis1) functioned better than the others (see results above and below). Therefore, differences in ability of the hybrids to complement functions cannot be explained solely by differences in protein levels.

**Figure 2:**
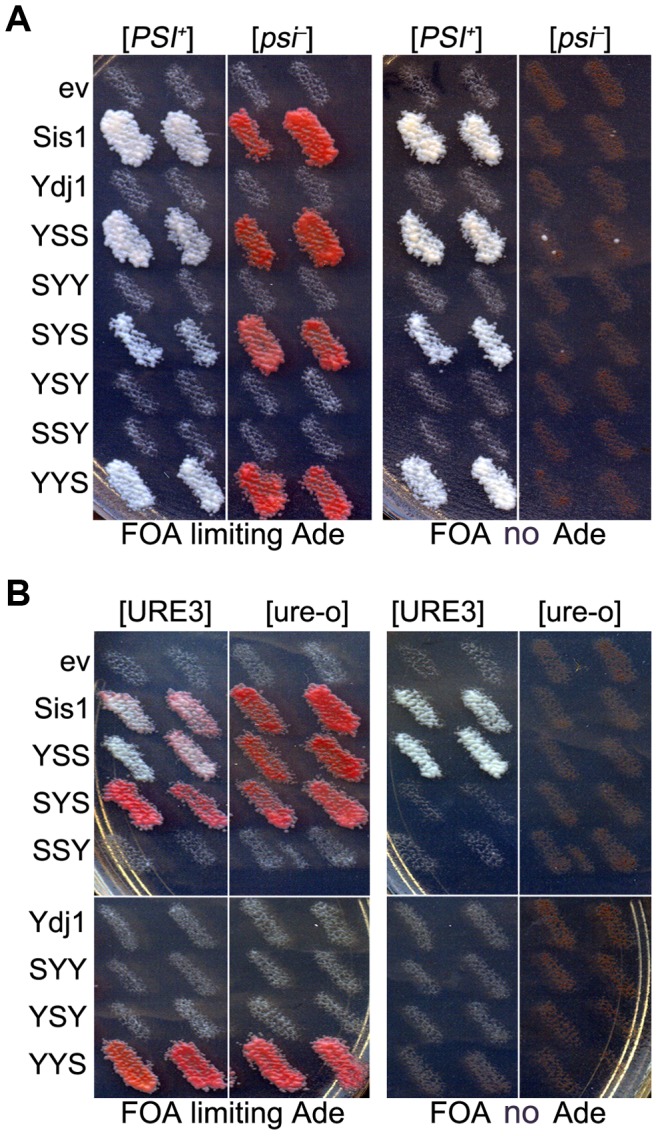
Functions of Sis1/Ydj1 hybrids in place of Sis1. (A) Growth and [*PSI^+^*] propagation: [*PSI^+^*] and [*psi^−^*] versions of strain 930 (*sis1Δ*) were transformed by plasmids encoding Sis/Ydj1 hybrid proteins. Transformants were grown as patches on medium containing uracil and then replica-plated onto FOA with (left panels) and without (right panels) adenine. Each patch is an individual transformant, similar results were obtained in two other transformations. (B) [URE3] propagation: [URE3] and [ure-o] versions of strain 1385 (*sis1Δ*) were transformed by the same plasmids and processed as in (A). Cells with prions are Ade^+^ and white while cells lacking prions require exogenous adenine and are red when grown on limiting adenine (see Materials and methods). In all panels each patch is from an independent transformant colony from one of three independent transformations. All of 3 replicated experiments gave the same results.

To determine if individual Sis1 sub-regions within full-length proteins are enough to support growth of *sis1Δ* cells, both [*PSI^+^*] and [*psi^−^*] versions of strain 930 (*sis1Δ* carrying wild type *SIS1* on a *URA3* plasmid) were transformed with plasmids encoding the hybrid proteins and then grown as patches on medium that allows cells to lose the *URA3* plasmid encoding wild type Sis1. These were then replica-plated onto medium containing FOA to select for cells having lost that plasmid. Regardless of prion status, all hybrids that contained the Sis1 CTD, and only these hybrids, supported growth (Figure 2A, left panels). Therefore, in the context of our full-length hybrids, the CTD of Sis1 was necessary and sufficient to provide essential Sis1 activity. These same hybrids supported propagation of [*PSI* ^+^], which is consistent with our earlier findings that propagation of [*PSI* ^+^] is minimally dependent on Sis1 and that any Sis1 mutant that supports growth also supports [*PSI* ^+^] [32].

As expected, these same hybrids supported growth of isogenic *sis1Δ* strain 1385 (used to monitor [URE3]). However, only those containing both the CTD and GF regions of Sis1 (i.e. Sis1 and YSS) supported propagation of [URE3] (Figure 2B). Thus, propagation of [URE3] depended on the GF/GM and CTD regions of Sis1, but not on the Sis1 J-domain. The Ydj1 GF region did not function in place of Sis1 GF/GM (i.e. in SYS) to support prion propagation. These latter observations are reminiscent of earlier work showing that a short stretch of the GF region in Sis1, which is absent in Ydj1, is important for propagation of [*PIN^+^*] [23], and they suggest the same function is important for propagation of [URE3].

### The CTD of Ydj1 supports Ydj1 functions

Aside from its role in protecting cells from exposure to lethal heat, Ydj1 is important for cell growth under all conditions [27]. Cells lacking Ydj1 are viable, but they grow very slowly at 25°C and do not grow at 34°C. Elevating expression of Sis1 and other J-proteins, or even J-domains alone, can improve growth of *ydj1Δ* cells [14], [34], which suggests that the functions of Ydj1 in its roles important for viability are more general. Nevertheless, we tested if distinct domains of Sis1 and Ydj1 conferred Ydj1-specific functions important for growth by repeating the plasmid shuffle using *ydj1Δ* strain MR502, which has *YDJ1* on a *URA3* plasmid.

**Figure 3:**
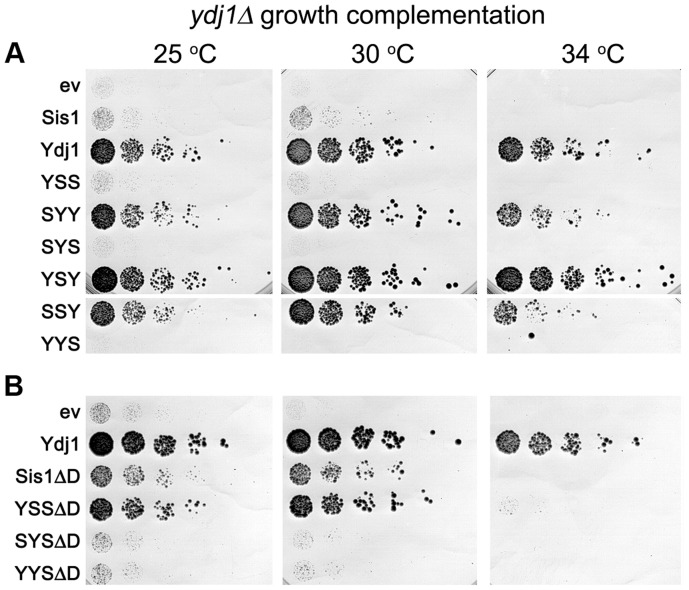
Growth complementation by Sis1/Ydj1 hybrids in place of Ydj1. (A) Transformants of strain MR502 (*ydj1Δ* with *YDJ1* on a *URA3* plasmid) expressing the indicated hybrid proteins from the *SIS1* promoter on single-copy plasmids (ev is empty vector) were taken from FOA plates and grown in liquid medium selecting for the plasmids. Cultures were normalized to the same cell density, serially diluted five-fold and dropped onto YPAD plates, which were incubated at the indicated temperatures for three days. (B) Cells expressing Sis1 or the hybrids with the Sis1 CTD that lack their dimerization domains (ΔD) were processed and grown as in (A).

We found that all hybrid proteins containing the CTD of Ydj1 restored growth noticeably, even at 34°C (Figure 3A). The substantial ability of YSY and SSY to restore growth indicates that the Ydj1 GF region is effectively dispensable for its functions in cell growth and its J-domain has a small contribution. Compared with the empty vector, Sis1 also improved growth weakly at 30°C, which is in line with earlier data showing increased expression of Sis1 compensates for loss of Ydj1 [27], but it did not support growth at 34°C (Figure 3A). Cells with YSS grew slightly better than those with the empty vector at 25°C and 30°C, but the other hybrids containing the Sis1 CTD failed to improve growth, even at 25°C. These results suggest that in the context of our full-length proteins the CTD of Ydj1 possesses Ydj1-specific functions important for growth.

As indicated above, increasing expression of proteins with the CTD of Sis1 inhibited growth of *ydj1Δ* cells in a dose-dependent manner (Figure S3). One explanation for this effect is that hybrids with the Sis1 CTD were able to form defective heterodimers with wild type Sis1 and a resulting impairment of Sis1 function would exacerbate the growth defect caused by lack of Ydj1. To test this possibility, we repeated the experiments using hybrids lacking the dimerization domain (ΔD). Even though the abundance of these truncated proteins was at the low level of their counterparts (Figure S2C), YSSΔD improved growth considerably at 30°C and allowed weak growth at 34°C (Figure 3B). SYSΔD and YYSΔD also supported growth at 30°C, but only slightly. These results are consistent with the growth inhibition being caused by dominant inactivation of wild type Sis1 and suggest that lack of complementation was not necessarily due to lower protein abundance.

Deleting the dimerization domain of wild type Sis1 also improved its ability to suppress the *ydj1Δ* defect, although not enough to support growth at 34°C. Thus, monomeric Sis1 is better at performing Ydj1-specific functions than Sis1 dimers. Together these results agree with earlier work showing a general ability of truncated J-proteins to complement Ydj1 function better than intact proteins [34]. They also suggest dimerization might specify or restrict Hsp40 activities.

**Figure 4:**
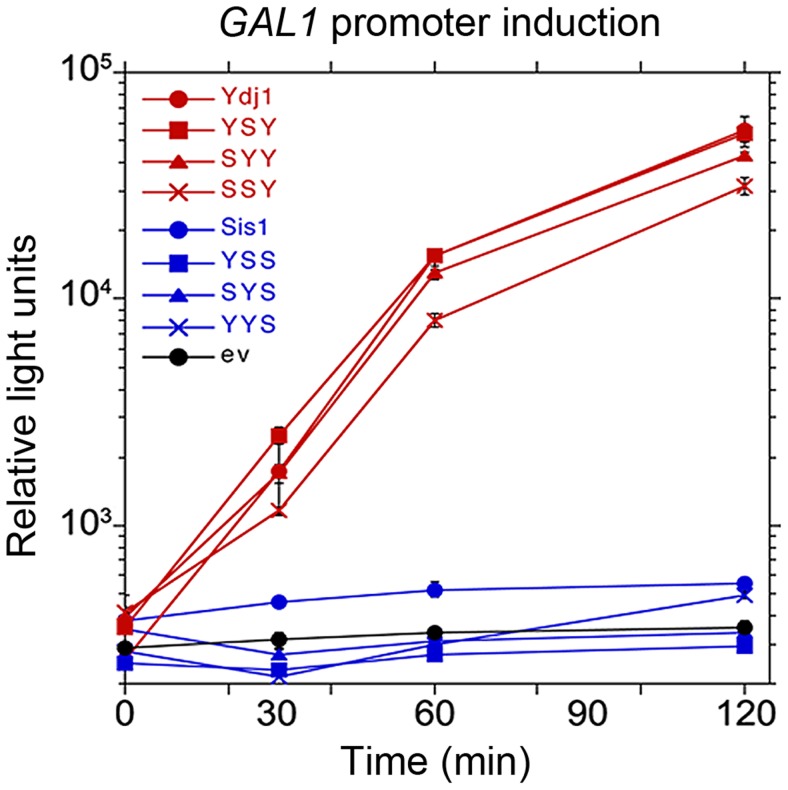
Function of Sis1/Ydj1 hybrids in galactose induction. Cells expressing indicated hybrid proteins (from Figure 3, panel A) were assessed for ability to induce expression of a luciferase reporter regulated by the *GAL10* promoter. After adjusting cultures to the same cell density, galactose was added and the luciferase activity in aliquots of cells was then measured periodically for two hours. Cells did not grow during this time.

Ydj1 is also a critical component of the Hsp90 molecular chaperone system necessary for activating galactose-inducible gene promoters [37]. This machinery is thought to remove nucleosomes from the promoter region to allow access to transcription factors. Deleting *YDJ1* abolishes galactose induction by disrupting this process. When assessed for ability to function in galactose induction (Figure 4), all of the hybrids that had the CTD of Ydj1, and only these hybrids, restored *GAL* expression. Thus, the CTD of Ydj1 determined specificity of Ydj1 in a process that involves its cooperation with the Hsp90 machinery.

### Hsp40 CTDs specify cooperation of purified chaperones

**Figure 5:**
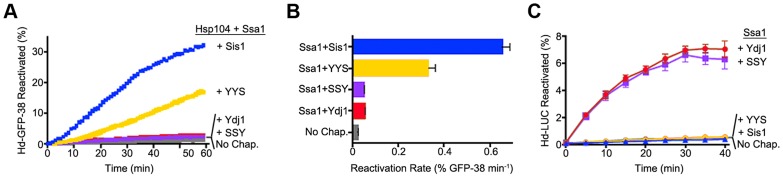
Function of Sis1, Ydj1 and Sis1-Ydj1 hybrid proteins in protein reactivation in vitro. (A) Reactivation of heat-denatured GFP-38 by Sis1 (blue), Ydj1 (red), SSY (purple) or YYS (yellow) in combination with Hsp104 and Ssa1 was measured over time as described in Methods. Denatured GFP-38 incubated without chaperones is shown in gray. A representative plot of 3 experiments is shown. (B) Initial rates of reactivation of heat-inactivated GFP-38 by Hsp104 and Ssa1 in conjunction with Sis1, Ydj1, SSY or YYS (n = 3, ± SEM). (C) Reactivation of heat-denatured luciferase by Sis1 (blue triangles), Ydj1 (red circles), SSY (purple squares) or YYS (yellow circles) in combination with Ssa1 was measured as described in Methods. Denatured luciferase incubated without chaperones is shown in black circles. Data from three replicates are presented as the mean ± SEM.

We next tested whether the ability of the Hsp40s to exhibit functional discrimination in vivo was reflected in discrimination in vitro. To do this we monitored protein reactivation of two different substrates. When heat-inactivated GFP-38, a GFP fusion protein containing a C-terminal 38 amino acid peptide, was used as the substrate, Sis1 in combination with Hsp104 and Ssa1 restored about 30% of the GFP-38 after an hour (Figure 5A). With Ydj1 in place of Sis1 in the reaction, there was little reactivation (<2%). The hybrid protein containing the CTD of Sis1, YYS, was able to reactivate GFP-38 with Hsp104 and Ssa1, but the rate of reactivation was ∼50% that of Sis1 (Figure 5A and B). There was no detectable reactivation of GFP-38 by SSY under the same conditions. These results show that reactivation of GFP-38 by Hsp104 and Ssa1 requires a Sis1-specific function and that the CTD of Sis1, when appended to the J-GF of Ydj1, was sufficient to provide this function.

In contrast, with heat-inactivated luciferase as substrate, Ydj1 in combination with Ssa1 promoted reactivation and Sis1 with Ssa1 was inactive (Figure 5C). SSY was as active as Ydj1 in reactivating luciferase with Ssa1. YYS in combination with Ssa1 was unable to reactivate heat-denatured luciferase. Together these results show that Sis1 and Ydj1 discriminate between protein aggregates and discrimination is a function of the Sis1 and Ydj1 CTDs.

### Sis1 mutants dominantly inhibit [URE3] propagation

**Figure 6:**
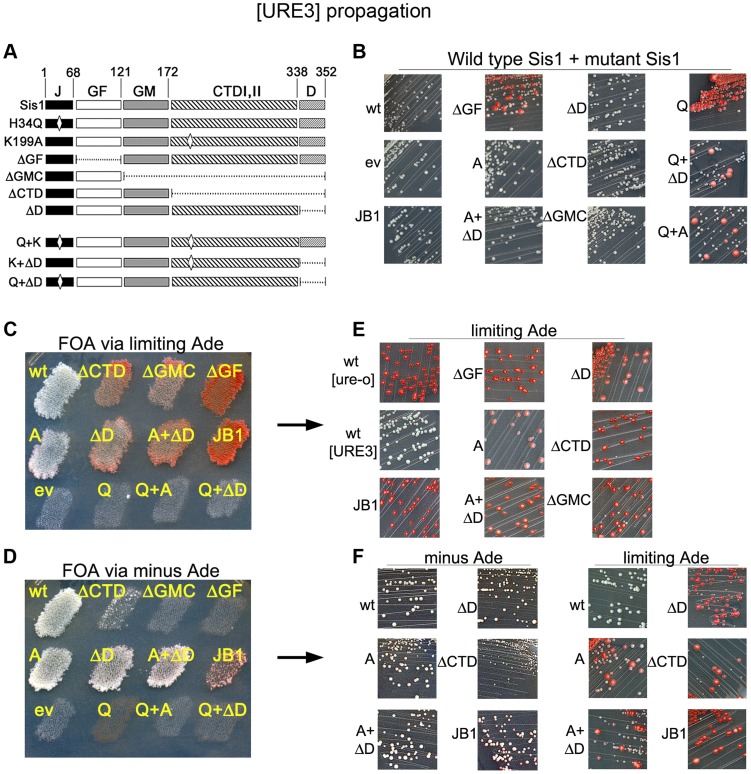
Sis1 activities are important for [URE3] propagation. (A) Diagram (not to scale) of Sis1 coding region. Numbers at top indicate amino acid positions. Domains, designated by abbreviations at top (see text) are indicated by variously shaded boxes. Mutations are indicated on the left. Deleted regions are shown as dashed lines and locations of point mutations H34Q and K199A as diamonds. Q is H34Q, A is K199A. Intact DnaJB1 (not diagrammed), is the closest human homolog of Sis1 and is indicated JB1 in the other panels. (B) Strain 1385 expressing wild type Sis1 from a *URA3* plasmid was transformed by single-copy *TRP1* plasmids encoding the wild type or mutant proteins (indicated on the left of each image) regulated by the *SIS1* promoter. Transformants were taken from plates selecting for [URE3] and both plasmids and streaked onto plates selecting only for the plasmids. Plates were incubated for 3 days at 30°C followed by 2 days at 25°C. [URE3] cells form small white colonies, while [ure-o] cells grow into larger red colonies. (C) [URE3] cells as in panel (B) were grown as patches on plates selecting for [URE3] and both plasmids. These were replica-plated onto medium containing uracil to allow loss of the *URA3* plasmid, and then onto medium containing limiting adenine and FOA (shown), which kills cells retaining the *URA3* plasmid. Empty vector control is indicated as ev. (D) As in (C) except adenine was omitted from all plates to ensure growth only of cells that propagate [URE3]. (E) Cells from plate in panel (C) were streaked onto similar medium. The fainter red coloration of [ure-o] cells expressing the ‘A’ mutant is due to a slightly higher amount of adenine in the medium. (F) Cells from plate in panel (D) were streaked onto medium containing or lacking adenine, as indicated. In all panels, red colonies arose from cells that lost [URE3].

The extent that [URE3] depends on Sis1 has not been evaluated systematically. We monitored [URE3] in *sis1Δ* strain 1385, which carries a *URA3*-based plasmid encoding Sis1 to support viability. We expressed previously described versions of Sis1 engineered to contain deletions or point mutations from a *TRP1*-based plasmid [32]. Deletions remove defined structural domains, the H34Q substitution in a conserved histidine-proline-aspartate (HPD) motif comprising residues 34–36 in the J-domain disrupts a critical interaction with Hsp70 [38], [39], and the K199A substitution in the CTD disrupts substrate binding [40] (see Figure 6A). To assess evolutionary conservation of Hsp40 function we also included the human Sis1 homolog DnaJB1 (also known as Hdj1). When expressed in place of Sis1, DnaJB1 supports cell viability and propagation of certain variants of [*PIN^+^*] and [*PSI^+^*] [23], [32], [41], [42]. Depleting functional Ure2 into [URE3] prion aggregates makes our strains grow slowly [15], which is evident when comparing sizes of [ure-o] and [URE3] colonies (see Figure 6B).

When Sis1 proteins lacking the GF region or containing the H34Q point mutation were expressed with wild type Sis1 they had obvious dominant inhibitory effects on [URE3], seen as appearance of red [ure-o] colonies (Figure 6B). Because the H34Q mutation disrupts physical interaction of J-proteins with Hsp70, the dominant inhibition of [URE3] propagation by the H34Q mutant might be caused by its forming defective hetero-dimers with wild type Sis1 or by competing with Sis1 for substrate.

To test these possibilities, we combined H34Q with alterations that interfere with ability of Sis1 to dimerize (ΔD) or to bind substrate (K199A). Both mutations reduced the dominant anti-[URE3] effect to a similar extent (from ∼23% to ∼6% [ure-o] colonies), but did not eliminate it (Figure 6B, rightmost images). Thus, inhibition of [URE3] by Sis1-H34Q depended partially on each of these Sis1 functions, suggesting it could be acting by making defective dimers with wild type Sis1 or by competing with Sis1 for binding to substrate, which in this system would be Ure2 amyloid. Although blocking dimerization can affect cooperative interaction of Sis1 with substrates in vitro [24], these results suggest that Sis1-H34Q can interfere with functions of wild type Sis1 in multiple ways.

### All Sis1 activities are important for [URE3] propagation

To determine if the mutant Sis1 proteins could support propagation of [URE3], we counter-selected against the *URA3* plasmid encoding wild type Sis1 as described in Figure 2A (see Figure 6C). Because several mutant Sis1 proteins appeared incapable of supporting [URE3] when this plasmid shuffling was done on plates without selecting for the prion, we also replica-plated the same patches of cells onto a series of similar plates lacking adenine to ensure recovery of cells capable of propagating [URE3], but weakly (Figure 6D). Cells will grow on FOA lacking adenine only if the Sis1 mutant supports both growth and [URE3] propagation.

Cells expressing each of the mutant Sis1 proteins, except those containing the lethal H34Q mutation, grew on the FOA plate that contained adenine (Figure 6C), showing the mutant proteins supported growth in place of Sis1. However, only the cells carrying wild type Sis1 had a normal white [URE3] phenotype on this plate, indicating [URE3] was lost rapidly from cells expressing any of the mutant Sis1 proteins as soon as the plasmid encoding wild type Sis1 was lost. Accordingly, when cells from this plate were streaked for isolated colonies on medium containing adenine, only the cells expressing wild type Sis1 gave rise to uniformly white [URE3] colonies (Figure 6E). Therefore, stable propagation of [URE3] depended on all of the Sis1 activities tested.

Although cells expressing Sis1ΔD, Sis1-K199A and the mutant with both of these mutations propagated [URE3] when selection for the prion was maintained (Figure 6D), they all lost [URE3] rapidly when grown on medium containing adenine (Figure 6F, right panels). On medium lacking adenine, [URE3] cells expressing most of the mutant proteins formed colonies at a rate similar to those expressing wild type Sis1p (Figure 6F, left panels), suggesting that the rapid loss of the prion under non-selective conditions was not due to the prion causing a disproportionate inhibitory effect on growth. However, it was evident that the Sis1ΔCTD [URE3] cells grew much more slowly than the others on medium selecting for [URE3] (Figure 6D, 6F, images on left). Since [ure-o] cells expressing Sis1ΔCTD grew like wild type [ure-o] cells (compare Figure 6E middle right image with upper left image) this slower growth was caused by the presence of [URE3], suggesting that the CTD region of Sis1 protects cells from toxic effects of [URE3]. Similar prion-associated toxicity was seen for [*PSI^+^*] cells expressing Sis1ΔCTD in place of Sis1 [32], [41].

We did not recover cells expressing Sis1ΔGF or Sis1ΔGMCTD on FOA plates lacking adenine. Thus, in agreement with results using the hybrid proteins, [URE3] requires the Sis1 GF region to propagate. The inability to recover [URE3] cells expressing Sis1ΔGMCTD might indicate [URE3] is even more toxic in these cells. Alternatively, [URE3] could be unable to propagate in cells expressing only JGF even under conditions selecting for the prion. DnaJB1 propagated [URE3] only under selective conditions, and even then only weakly. Overall, our results indicate that [URE3] depends much more on Sis1 than [*PSI^+^*] does.

We showed earlier that BKE (with wild type DnaK) supports [*PSI^+^*] propagation [15], so we tested if this system would also be useful for studying [URE3]. BKE did not support [URE3] in *hsp104Δ* cells (Figure S4). Because wild type DnaK should be able to interact with J-proteins other than Sis1, any or all of the cytosolic J-proteins might be able to compete with Sis1 for interaction with DnaK. Since [URE3] has a stringent requirement for Sis1, a resulting reduction in ability of Sis1 to interact with the BKE machinery probably explains the inability of [URE3] to be propagated. Alternatively, as stable propagation of [URE3] depends critically on which Hsp70 is present [43], [44], the failure might reflect a requirement for a specific Hsp70 activity lacking in DnaK.

### Sensitivity of [URE3] to Sis1 alteration explains how Ydj1 cures [URE3]

Although the underlying mechanism of how overexpressed Ydj1 cures cells of [URE3] is uncertain, interaction with Hsp70 is critical because Ydj1 mutants unable to interact with Hsp70 do not cure and the J-domain alone of Ydj1 or other yeast Hsp40s is enough to cure [14], [45]. Since all parts of Ydj1 except the J-domain can be mutated or deleted without disrupting curing, we expected the CTD of Ydj1 would not have a major influence on the curing of [URE3]. Instead, we anticipated that if a hybrid cannot function in place of Sis1 with the disaggregation machinery that replicates [URE3] prions, then it will interfere with this function if it can compete effectively with Sis1 for interaction with the Hsp70 component of this machinery.

**Figure 7:**
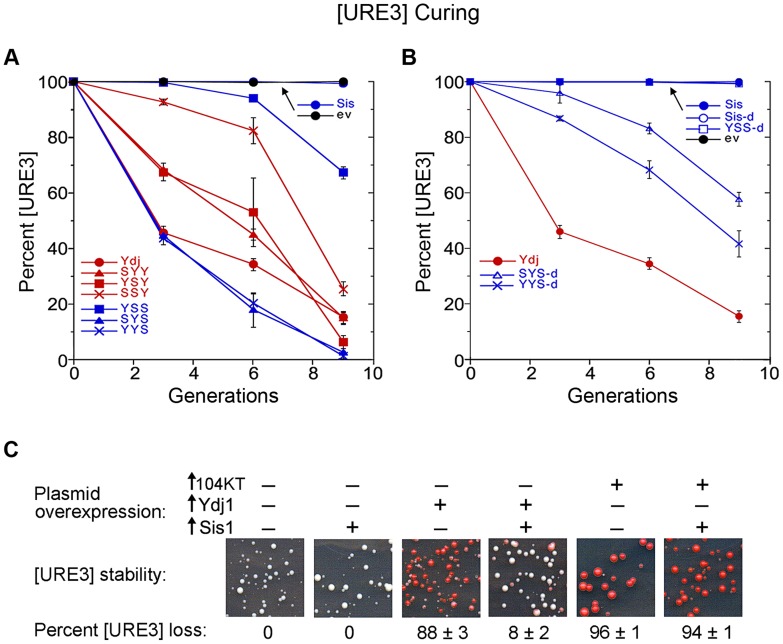
Curing of [URE3] by overexpression of Ydj1 and hybrid proteins. (A) Wild type [URE3] cells (strain 1075) carrying plasmids encoding indicated hybrid proteins under control of the *GAL* promoter were grown in dextrose medium selecting for the plasmid, washed, and transferred to galactose medium. The proportion of [URE3] cells as a function of culture generations in galactose is shown. (B) Curing was done simultaneously using identical conditions except Sis1 and hybrid proteins lacking their dimerization domains (open symbols, as indicated) were used. For easier comparison, these data are plotted separately with the same Ydj1 data. (C) Elevating abundance of Sis1 reduces curing of [URE3] by overexpressing Ydj1, but not Hsp104. Strain 1075 was transformed simultaneously by indicated combinations of empty vectors (−) and single-copy plasmids expressing Ydj1, dominant negative Hsp104-2KT or Sis1 (+) from the GPD promoter. Images show sections of representative primary transformation plates after incubating 4 days at 30°C. Numbers below images indicate average frequency of [URE3] loss (± s.d.).

When overexpressed, SYS and YYS cured like Ydj1 (see Figure 7A). SYY and YSY cured somewhat less effectively, and YSS cured inefficiently. Thus, the SYS and YYS hybrids that did not propagate [URE3] cured [URE3] very effectively, while YSS, which supported [URE3], cured only weakly.

Since SYS and YYS possess the dimerization region of Sis1, their ability to cure [URE3] when overexpressed again might be related to an ability to form non-productive dimers with endogenous Sis1, which could contribute to the curing by partially depleting cytosolic Sis1 function. In agreement with this explanation, although disrupting dimerization of Ydj1 does not affect curing considerably [45], hybrids with the CTD of Sis1 that lacked the dimerization domain were significantly reduced in their ability to cure [URE3] (Figure 7B). The residual curing by monomeric SYS and YYS could be explained by their competing with endogenous Sis1 for interaction with Hsp70 or with [URE3] as a substrate.

Our curing data add to much previously published work [14], [15], [23], [45], [46] that support the explanation that overexpressing Ydj1 cures [URE3] by competing with Sis1. If so, then increasing abundance of Sis1 should allow it to compete more effectively for the disaggregation machinery and reduce the curing. In line with this prediction, overexpressing Ydj1 cured [URE3] much less effectively in cells with elevated expression of Sis1 (Figure 7C).

It remained possible that elevating Sis1 reduced this curing through some general stabilizing effect on [URE3]. However, if increasing Sis1 protects [URE3] from Ydj1 curing specifically by improving ability of Sis1 to compete with Ydj1, then increasing Sis1 would not be expected to protect [URE3] from being cured by other ways of impairing disaggregation machinery activity, such as inhibiting Hsp104. Overexpressing the dominant negative Hsp104-2KT mutant [5], which inhibits Hsp104 activity, cured [URE3] very effectively (Figure 7C). Elevating Sis1 expression did not affect this curing. Thus, Sis1 specifically counteracted curing by overexpressed Ydj1, which again is consistent with the idea that Ydj1 cures [URE3] by competing with Sis1 for interaction with the disaggregation machinery.

### [*PIN^+^*] is less dependent on Sis1 functions than [URE3]

**Figure 8:**
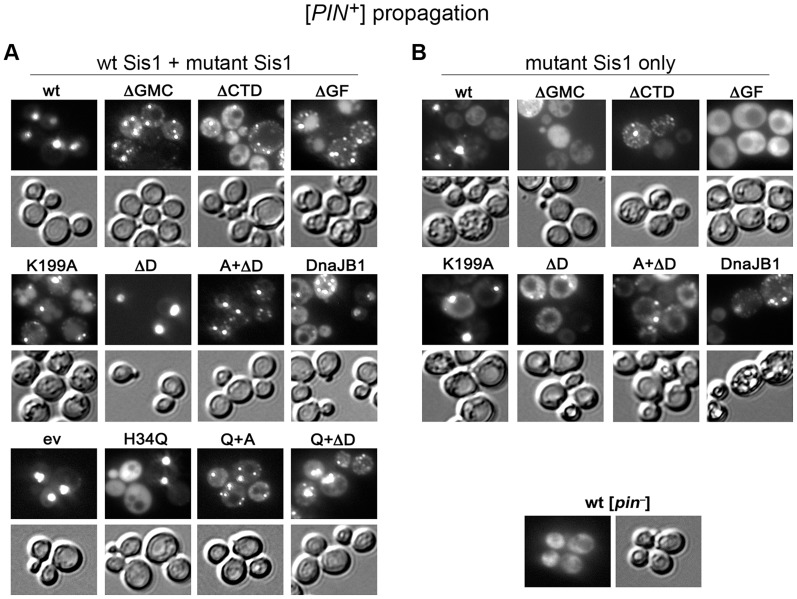
Some Sis1 activities are important for [*PIN^+^*] propagation. [*PIN^+^*] [*psi^−^*] cells of strain 930a expressing Rnq1-GFP were transformed by plasmids encoding the mutant Sis1 proteins described in Figure 5. (A) Primary transformant colonies on medium selecting for the plasmids encoding both wild type and mutant Sis1 proteins were assessed for fluorescence. (B) Cells shown in panel (A) were transferred to plates containing uracil and then onto FOA plates to select for cells expressing the only mutant versions of Sis1. Cells from the FOA plates were then assessed for fluorescence. For each set, upper panels are fluorescent images and lower panels show the same cells in bright field. Control [*pin^−^*] cells carrying one copy of wild type Sis1 are shown on lower right for comparison.

We repeated the plasmid shuffle in [*psi^−^*] [*PIN^+^*] strain 930a to assess effects of our panel of Sis1 mutants on propagation of [*PIN^+^*] prions (Figure 8). We monitor [*PIN^+^*] by the fluorescence status of Rnq1-GFP, which is regulated by the *RNQ1* promoter on a single-copy plasmid. Rnq1-GFP is punctate in [*PIN^+^*] cells and diffuse in [*pin^−^*] cells.

[*PIN^+^*] propagated in cells with wild type Sis1 regardless of which of the mutant proteins was also expressed (Figure 8A). However, while wild type cells had single bright foci, those co-expressing the mutant proteins, except for Sis1ΔD and H34Q, had multiple foci (multi-dot). Therefore, several mutant Sis1 proteins dominantly affected propagation of [*PIN^+^*]. Cultures expressing Sis1-H34Q had a mixture of cells that possessed either single foci or completely diffuse fluorescence, indicating that H34Q inhibited the wild type Sis1 enough to cause [*PIN^+^*] to be lost from some cells. Combining ΔD or K199A with the H34Q mutation led to a multi-dot phenotype and reduced the proportion of [*pin^−^*] cells. These effects resemble the way the Sis1 mutants dominantly inhibited [URE3] and again show that the prion-curing effect of Sis1-H34Q depends partially on its dimerization and substrate-binding functions. Unlike [URE3], [*PIN^+^*] was not dramatically destabilized by co-expression of Sis1ΔGF, here regulated by the *SIS1* promoter on a single copy plasmid. However, overexpressing Sis1ΔGF in [*PIN^+^*] cells of another strain background is toxic and causes [*PIN^+^*] to be lost [47].

In agreement with earlier work [23], [30], Sis1ΔCTD supported [*PIN^+^*] in cells without wild type Sis1, but Sis1ΔGF did not (Figure 8B). However, unlike the earlier work that showed Sis1ΔGMCTD (i.e. Sis1 JGF alone) propagated [*PIN^+^*], we did not observe [*PIN^+^*] foci in cells expressing Sis1ΔGMCTD. This difference might be due to differences in yeast strain backgrounds or by our variant of [*PIN^+^*] being more dependent on Sis1 for its propagation. In an earlier study the single-dot character of [*PIN^+^*] aggregates was frequently transformed by Sis1ΔGMCTD to multiple-dots, which were inheritable after transfer to wild type cells [30]. It is possible that the altered Sis1 activity causing this change is related to the loss of [*PIN^+^*] in our strains expressing Sis1ΔGMCTD, or that the action of Sis1ΔGMCTD on [*PIN^+^*] might be different in the two yeast strain backgrounds due to variation in expression of other chaperones.

[*PIN^+^*] also propagated stably enough to be detected among most cells expressing the substrate-binding and dimerization mutants (Figure 8B), showing that while these functions are important for [*PIN^+^*] propagation, [*PIN^+^*] depended less on these Sis1 activities than [URE3]. Keeping in mind that variations among strains of yeast and prions can influence prion stability, our data showing this intermediate sensitivity of [*PIN^+^*] to impairment of Sis1 activity is consistent with it being less sensitive than [URE3], but more sensitive than [*PSI^+^*], to curing by overexpressed Ydj1. Finally, we confirm earlier findings that DnaJB1 (Hdj1) supports propagation of [*PIN^+^*] [30], [32], [41].

## Discussion

Hsp40s bind misfolded proteins and regulate Hsp70 activity, so the ability of Sis1 and Ydj1 to specify functions of the disaggregation machinery are likely to be mediated through interactions with substrate and Hsp70. Our findings here show that C-terminal domains of Sis1 and Ydj1 can determine their functional differences in prion propagation, thermotolerance, galactose induction and their specific and general roles in supporting cell growth. Because the primary sites in Sis1 and Ydj1 that interact with substrates are contained within the CTDs, our data support the view that functional distinctions among Hsp40s can be due to differences in substrate specificity [34], [35].

The CTD of Sis1 also interacts with Hsp70, however, [47], [48] and although not fully characterized, this interaction likely influences Hsp70 functions. Likewise, functions influenced by the zinc-finger and farnesylation of the CTD of Ydj1 are important for the transfer of substrate to Hsp70 and for protecting cells from a [*PIN^+^*] prion-related toxicity [49], [50]. Also, Ydj1 can interact physically with Hsp104 in vitro [2], although the relevance of this interaction in the cell is unclear. While these other activities can be expected to contribute to specificity of these Hsp40s, our in-vivo and in vitro results indicate that the CTDs alone of Sis1 and Ydj1 allow them to discriminate between specific substrates, which is in line with earlier data [35].

A plausible explanation for the functional distinctions we observe would be that the CTD of Ydj1 interacts more readily with amorphous aggregates of stress-denatured proteins while that of Sis1 targets the more structured and homogeneous prion polymers. Sis1 and Ydj1 both bind to prion proteins, although Sis1 seems to bind more avidly, and prion proteins can differ in the number or location of general and distinct binding sites recognized by different Hsp40s [50]–[54]. Additionally, because substrate specificity of Hsp40s can overlap, competition among Hsp40s for substrates could contribute to determining functions of the chaperone machinery.

As seen earlier [47], we found the GF region can confer prion-specific Hsp40 functions. We show that the GF region of Sis1 was needed to propagate [URE3]. [*PIN^+^*] also depends on an activity of the Sis1 GF region that cannot be complemented by the Ydj1 GF [22], [23], [30]. However, all testable activities of Sis1 are dispensable for [*PSI^+^*] propagation [32], which shows that functions of the Sis1 GF are not necessary for propagation of all prions. Nevertheless, when appended to the JGF of their counterparts, the CTDs of Ydj1 and Sis1 generally were enough to allow the hybrid proteins to perform distinctly and effectively in place of intact Ydj1 and Sis1. Thus, the J and GF regions of Sis1 and Ydj1 possess activities that overlap enough to perform similarly in several distinct tasks. Evidently, more work is needed to learn how the GF region specifies Hsp40 functions in its effects on prions and perhaps other Hsp40-dependent cellular processes.

Much evidence points to Sis1 playing a key role in the replication of yeast prions by acting as a component of the Hsp104 disaggregation machinery that fragments prion fibers [14], [30], [46], [55], [56]. The varying degrees by which the prions depend on Sis1 agree with the supposition that different prions, and even different strains of the same prion (see [46], [57]), require varying degrees of disaggregation machinery activity to be fragmented. Together with the insensitivity of [*PSI^+^*] to Sis1 mutation, our finding that [URE3] is acutely sensitive to alteration in any Sis1 activity helps explain why depleting Sis1 causes cells to lose [URE3] much faster than they lose [*PSI^+^*] [14]. Our finding that [*PIN^+^*] had an intermediate dependency on Sis1 activity is also consistent with the intermediate rate of loss of [*PIN^+^*] seen upon Sis1 depletion. Overall our findings are consistent with earlier suggestions of a hierarchical dependency of these and other prions on the disaggregation machinery [57].

Extending this reasoning, our data strongly support an earlier suggestion that curing of [URE3] by Ydj1 or J-domains alone might be a result of competition for interaction with Hsp70 [14]. If Ydj1 cannot cooperate effectively with the disaggregation machinery to propagate [URE3], then by displacing Sis1 from the Hsp70 component of this machinery, less Hsp104 would be directed toward fragmenting prion polymers. This mechanism also explains why J-domains alone are enough to destabilize [URE3] and aligns with the idea that certain intact J-proteins don't cure as effectively because they are normally recruited to defined locations in the cytosol, such as ribosomes, by their other distinct functional domains. Among the three prions tested [URE3] is most sensitive to reductions in Sis1 function, so one might expect that its propagation would be most affected by such competition.

In line with a more stringent requirement of the disaggregation machinery for [URE3] replication, the average number of [URE3] prions per cell is lower than that for [*PIN^+^*] and [*PSI^+^*] [14], [58], [59]. The different seed numbers among variants of [*PIN^+^*] also could reflect differences in susceptibility to fragmentation, which in turn might underlie variation in sensitivity to curing by overexpressed Ydj1 [29]. Differences in susceptibility to fragmentation can be due to subtle differences in the structures of the amyloid that form the prions [60]. Such variation in the amyloid structures that determine differences among variants of [*PIN^+^*] might also have a bearing on the distinct pattern of variants of [*PSI^+^*] they induce [29], [61]–[63]. The similar intermediate sensitivity of [*PIN^+^*] to depletion of Sis1 seen in earlier work and to specific mutations of Sis1 seen here suggests the variants of prions used are similar and that their prion character is largely independent of strain background. Nevertheless, findings might differ if other variants of prions or other strain backgrounds propagating them were compared directly.

It is becoming evident that the differences in ways prions respond to J-proteins and other Hsp70 co-chaperones likely reflect differences in the ways prions depend on Hsp70. Altering activity of Hsp70 directly by mutation or indirectly by altering its co-chaperones can influence prion propagation in the same ways, which supports this idea [64]–[66]. Stability of [*SWI^+^*] prions is also highly sensitive to altered expression of Hsp40s and J-domains, which seems to be related to a strict dependency on optimal Hsp70 activity [67]. Yeast has four highly homologous Ssa Hsp70 paralogs and prion phenotypes vary greatly when different Hsp70s are the sole source of Ssa protein [43]. These differences likely reflect differences in the way the Hsp70s interact with or are regulated by the Hsp40s or other factors. Hsp70 also can be a primary factor in recruiting the disaggregation machinery to prion polymers [7]. Notably, however, it is not Hsp70 substrate-binding *per se*, but the regulation of this binding, presumably by co-chaperones, that specifies distinctions in Hsp70 functions with regard to [URE3] propagation [44]. NEFs can also affect prion propagation through their ability to regulate Hsp70 [13], [68], [69].

Because Hsp70 is a critical component of the Hsp104-based disaggregation machinery, altering Hsp70 or its co-chaperones can be expected to affect propagation of prions by influencing composition and activity of this machinery. The distinct susceptibilities of prions to alterations in various disaggregation machinery components might therefore reveal differences in the ways various chaperones combine to act most effectively on them as specific substrates. Understanding why prions respond differently to the various chaperone machinery components including J-proteins, NEFs and Hsp70s should help us understand both fundamental and subtle ways that these components interact to produce effective protein remodeling machines.

## Materials and Methods

### Yeast strains, plasmids, media and growth conditions

Yeast strains used are isogenic to strain 779-6A (*MATa*, *SUQ5*, *kar1-1*, *ade2-1*, *his3Δ202*, *leu2Δ1*, *trp1Δ63*, *ura3-52*) [70], which is used for monitoring [*PSI^+^*] and [*PIN^+^*]. Knockouts and replacements of chromosomal genes were done using standard transformation procedures [71]. Strain MR386 has *E. coli CLPB* in place of the chromosomal *HSP104* gene [15] and contains plasmids expressing *E. coli* dnaK^R167H^ (pMR150LG-R167H) and *E. coli* GrpE (pMR134H) under the control of the GPD (glyceraldehyde-3-phosphate dehydrogenase - *TDH3*) and *FES1* promoters, respectively. [*PSI* ^+^] is maintained in strain MR386 by pJ312, which is *HSP104* on a *URA3* plasmid [72]. Strain MR502 has *ydj1::KanMX* and carries p316YDJ1, which is *YDJ1* on a *URA3* plasmid. [*PSI^+^*] [*PIN^+^*] strain 930 has *sis1::KanMX* and carries plasmid pYW17, a *URA3*-based plasmid encoding wild type *SIS1* [22], [32].

Strain 930a is a [*psi^−^*] [*PIN^+^*] version of 930 that carries plasmid p313Rnq1-GFP. It was cured of [*PSI^+^*] by transient growth on medium containing 3 mM guanidine and then [*PIN^+^*] clones among [*psi^−^*] isolates were identified by punctate Rnq1-GFP fluorescence. Our [*PIN^+^*] variant is uncharacterized, but of the single-dot type, which typically has sturdier fibers and a lower seed number per cell than multi-dot [*PIN^+^*] [61]. Isogenic strain 1075, for monitoring [URE3], has *ADE2* regulated by the *DAL5* promoter (*P_DAL5_::ADE2*, see below) in place of *ade2-1* [43]. Strain 1385 is strain 1075 with *sis1::KanMX* and plasmid pYW17, which is *SIS1* on a *URA3* plasmid. Strains 1408 and 1410 are *hsp104Δ* versions of strain 779-6A and 1075, respectively [15]. Both carry pJ312. Our parental strains carry only one variant of [*PSI^+^*], [URE3] or [*PIN^+^*].

*SIS1* plasmids used contain wild type and mutant *SIS1* alleles on the pRS314 single-copy *TRP1* vector [22], [32]. Plasmid pRU4 is *LEU2*-based single-copy plasmid pRS415 containing the *GAL1* promoter and *CYC1* terminator flanking the polylinker sites *Spe*I and *Xho*I. For Gal-induced expression, *YDJ1* and hybrid alleles were inserted into pRU4 as *Bam*HI-*Sal*I fragments. All plasmids used in this study are listed in Table 1. Plasmids encoding *E. coli* genes or yeast Hsp40 genes with D36N mutations are described [15].

**Table 1:** Plasmids.

Plasmid	Description^a^	Marker	Source
pRS313	empty vector	*HIS3*	[82]
pRS314	empty vector	*TRP1*	[82]
pRS315	empty vector	*LEU2*	[82]
pRS316	empty vector	*URA3*	[82]
pYW17	*SIS1* (wild type)	*URA3*	[22]
pYW65	*SIS1* (wild type)	*TRP1*	[22]
pYW116	*sis1Δ71-121* (ΔGF)	*TRP1*	[22]
pYW66	*sis1Δ171-352* (ΔCTD)	*TRP1*	[22]
pYW62	*sis1Δ121-35*2 (ΔGMCTD)	*TRP1*	[22]
pYW118	*sis1^H34Q^* (Q)	*TRP1*	[22]
pGCH1	*sis1^K199A^* (K/A)	*TRP1*	[32]
pAK1	*sis1*Δ*338-352* (ΔD)	*TRP1*	[32]
pAK50	*DNAJB1* (human *SIS1*)	*TRP1*	[32]
pAK64	*sis1^H34Q,K199A^* (Q+K/A)	*TRP1*	[32]
pAK17	*sis1^H34Q^Δ338-352* (Q+ΔD)	*TRP1*	[32]
p313Rnq1-GFP	*RNQ1-GFP*	*HIS3*	[83]
pMR134H	*P_FES1_*::*GrpE* (*E. coli* NEF)	*HIS3*	This study
pMR150LG-R167H	*P*_GPD_::*dnaK^R167H^* (*E. coli* Hsp70)	*LEU2*	[15]
pMR169	*P_GAL10_*::Luciferase	*HIS3*	This study
pMR266	*P_SIS1_*::*SIS1-cmyc* (wild type)	*TRP1*	This study
pMR266ΔD	*P_SIS1_*::*sis1-ΔD-cmyc*	*TRP1*	This study
pMR267	*P_SIS1_*::*YDJ1-cmyc* (wild type)	*TRP1*	This study
pMR267-D36N	*P_SIS1_*::*ydj1^D36N^-cmyc*	*TRP1*	This study
pMR274	*P_SIS1_*::*YSS-cmyc*	*TRP1*	This study
pMR274ΔD	*P_SIS1_*::*YSSΔD-cmyc*	*TRP1*	This study
pMR275	*P_SIS1_*::*SYY-cmyc*	*TRP1*	This study
pMR276	*P_SIS1_*::*SYS-cmyc*	*TRP1*	This study
pMR276ΔD	*P_SIS1_*::*SYSΔD-cmyc*	*TRP1*	This study
pMR277	*P_SIS1_*::*YSY-cmyc*	*TRP1*	This study
pMR278	*P_SIS1_*::*SSY-cmyc*	*TRP1*	This study
pMR279	*P_SIS1_*::*YYS-cmyc*	*TRP1*	This study
pMR279ΔD	*P_SIS1_*::*YYSΔD-cmyc*	*TRP1*	This study
pMR286	*P*_GPD_::*SIS1-cmyc*	*TRP1*	This study
pMR286-D36N	*P*_GPD_::*sis1^D36N^-cmyc*	*TRP1*	This study
pMR286-D36N-ΔD	*P*_GPD_::*sis1^D36N^-ΔD-cmyc*	*TRP1*	This study
pMR287	*P*_GPD_::*YDJ1-cmyc*	*TRP1*	This study
pMR287-D36N	*P*_GPD_::*ydj1^D36N^-cmyc*	*TRP1*	This study
pMR288	*P*_GPD_::*YSS-cmyc*	*TRP1*	This study
pMR288-D36N	*P*_GPD_::*YSS^D36N^-cmyc*	*TRP1*	This study
pMR289-D36N	*P*_GPD_::*SYY^D36N^-cmyc*	*TRP1*	This study
pMR290	*P*_GPD_::*SYS-cmyc*	*TRP1*	This study
pMR290-D36N	*P*_GPD_::*SYS^D36N^-cmyc*	*TRP1*	This study
pMR291-D36N	*P*_GPD_::*YSY^D36N^-cmyc*	*TRP1*	This study
pMR292-D36N	*P*_GPD_::*SSY^D36N^-cmyc*	*TRP1*	This study
pMR293	*P*_GPD_::*YYS-cmyc*	*TRP1*	This study
pMR293-D36N	*P*_GPD_::*YYS^D36N^-cmyc*	*TRP1*	This study
pMR293-D36N-ΔD	*P*_GPD_::*YYS^D36N^-ΔD-cmyc*	*TRP1*	This study
pMR294	*P*_GPD_::*SIS1*	*LEU2*	This study
pMR306	*P*_GPD_::*hsp104^K218T,K620T^*	*TRP1*	This study
pMR315	*P*_GPD_::*sis1^D36N^Δ171-352* (ΔCTD)	*TRP1*	This study
pRU4	*P_GAL_* (empty vector)	*LEU2*	This study
pRU16	*P_GAL_*::*SIS1-cmyc* (wild type)	*LEU2*	This study
pRU20	*P_GAL_*::*YDJ1-cmyc* (wild type)	*LEU2*	This study
pRU36	*P_GAL_*::*YSS-cmyc*	*LEU2*	This study
pRU37	*P_GAL_*::*SYY-cmyc*	*LEU2*	This study
pRU38	*P_GAL_*::*SYS-cmyc*	*LEU2*	This study
pRU39	*P_GAL_*::*YSY-cmyc*	*LEU2*	This study
pRU40	*P_GAL_*::*SSY-cmyc*	*LEU2*	This study
pRU41	*P_GAL_*::*YYS-cmyc*	*LEU2*	This study
pRU42	*P_GAL_*::*sis1-ΔD-cmyc*	*LEU2*	This study
pRU43	*P_GAL_*::*YSS-ΔD-cmyc*	*LEU2*	This study
pRU44	*P_GAL_*::*SYS-ΔD-cmyc*	*LEU2*	This study
pRU45	*P_GAL_*::*YYS-ΔD-cmyc*	*LEU2*	This study
pJ312	*HSP104*	*LEU1*	[72]

a See Materials and Methods section for details of construction. All plasmids are *CEN* (single copy) unless otherwise indicated with “2-micron” (high copy). All pMR and pRU plasmids contain the *CYC1* terminator sequence immediately downstream of the ORF.

With the exceptions that 1/2YPD plates contain 5 g/L yeast extract, YPAD plates contain 400 mg/L (excess) adenine and solid defined media contain 10 mg/L (limiting) adenine, standard media and growth conditions were used [71].

### Construction of *SIS1-YDJ1* hybrid alleles

Sis1 and Ydj1 have clearly defined and characterized J-domains, glycine-phenylalanine (GF) rich middle domains, and a C-terminal region that contains two major elements (CTDI and CTDII) and a dimerization domain. The main structural differences between Sis1 and Ydj1 are a glycine-methionine-rich (GM) extension of the GF domain in Sis1, which has GF-redundant functions, and a Zn-finger domain (ZF) embedded between beta-strands 2 and 3 of the CTDI of Ydj1 that is absent in Sis1 [73]. Because the ZF of Ydj1 is an integral part of CTDI, we designed our Sis1-Ydj1 hybrids using the GF-CTDI junction to restrict the number of domain swaps and avoid using complicated junctions to swap the ZF region. Rather than designating the GM region as a separate domain, we combined the functionally redundant GF and GM regions of Sis1 into a single domain. Thus, hybrid alleles were made by swapping three regions: the J-domain, the glycine-rich region and the C-terminal portion, which includes CTDI, CTDII and the dimerization domains, herein referred to simply as the CTD (see Figure 1A, [30]). Hybrid genes were synthesized by GENEWIZ, Inc (South Plainfield, NJ) and sub-cloned into variants of pRS414 that placed the ORF under the control of the *SIS1*, *YDJ1* or GPD promoters and a downstream *CYC1* transcriptional terminator [74]. All constructs contained a c-terminal c-myc tag that had no noticeable affect on functions in vivo.

### Monitoring prions

Depletion of the ribosome release factor Sup35 by its sequestration in [*PSI^+^*] prion aggregates causes nonsense suppression. We monitored [*PSI^+^*] by suppression of the *ade2-1* nonsense allele in our strains. [*PSI^+^*] cells are Ade^+^ and white, while [*psi^−^*] cells are Ade^−^ and when grown on limiting adenine are red due to accumulation of a metabolite of adenine biosynthesis. The presence of [URE3] was monitored similarly by use of an *ADE2* allele regulated by the *DAL5* promoter (*P_DAL5_::ADE2*) [75], [76]. Under standard growth conditions Ure2 represses transcription of nitrogen metabolic genes, such as *DAL5*. [URE3] sequesters Ure2 into prion aggregates, thereby reducing Ure2 function and activating the *DAL5* promoter. Thus, [URE3] cells are Ade^+^ and white, while [ure-o] cells are Ade^−^ and red on limiting adenine. We confirmed that Ade^+^ phenotypes were due to the presence of prions by their guanidine curability and by crosses with cells lacking prions to produce a dominant, guanidine-curable Ade^+^ phenotype. We monitored [*PIN^+^*] by assessing aggregation status of a plasmid-expressed Rnq1-GFP fusion protein. GFP fluorescence is diffuse in [*pin^−^*] cells, but noticeably punctate in [*PIN^+^*] cells. In this study we used a typical strong [*PSI^+^*] strain and single variants of [URE3] and [*PIN^+^*] prions.

### Microscopy

Microscopic analysis of Rnq1-GFP fluorescence in live cells was done with a Nikon E-800 microscope with log phase cells grown in medium selecting for the plasmid encoding the Rnq1-GFP fusion protein. Images were captured using IVision software and processed using Adobe Photoshop software.

### Thermotolerance

Log phase cells grown in medium selecting for plasmids were diluted in fresh medium to an OD_600_ of 0.25 and 100 µL was transferred to 0.5 mL test tubes and placed in a PCR machine for thermocycling as indicated. At various times aliquots were removed and placed on ice. Cooled cells were serially diluted and 5 µL drops were spotted onto YPAD plates.

### Complementation assays

Strains 930 and 1385 (both *sis1Δ*) and derivatives of MR502 (*ydj1Δ*) carrying wild type *SIS1* or *YDJ1* on *URA3*-based plasmids were transformed by *TRP1*-based plasmids carrying wild type, mutant or hybrid alleles. Strain MR502 also carries pMR169 for monitoring *GAL* induction (see below). Transformants were grown as patches of cells on medium lacking both tryptophan and uracil and then replica-plated onto similar medium containing uracil to allow loss of the *URA3* plasmid. These were then replica-plated onto medium containing 5-FOA, which kills cells that did not lose the resident *URA3* plasmid.

For Sis1, growth on 5-FOA plates shows complementation of functions essential for growth, and growth without adenine shows complementation of Sis1 functions required for prion propagation. To test complementation of Ydj1 function, 5-FOA resistant cells of strain MR502 were grown overnight in medium selecting for the *TRP1* plasmids, normalized to the same cell density (OD_600_ = 0.25) and five-fold serially diluted. Aliquots of the dilutions (5 µL) were then dropped onto YPAD plates. Scanned images of the plates were taken after they were incubated at the indicated temperatures for 3–4 days.

### Galactose induction

Aliquots of overnight cultures of MR502 transformants used for growth complementation were transferred to synthetic raffinose medium (SRaf) and grown overnight. These cells carried the *TRP1*-marked hybrid alleles or empty vector control and a *HIS3*-marked plasmid encoding firefly luciferase under the control of the *GAL10* promoter (pMR169). Cell densities were adjusted to OD_600_ = 0.3 in fresh medium and the initial reading (t = 0) was taken by mixing 100 µL culture with 50 µL of 1 mM luciferin in 0.1 M sodium citrate, pH 5.0 immediately before reading in a Zylux Femtomaster luminometer, with a 10 s delay and 5 s read time. Galactose from a 20% stock was then added to the cultures to obtain a final concentration of 2% and the cells were incubated on a roller at 30°C. Readings were taken at 30, 60 and 120 minutes after addition of galactose. All samples in three experiments were tested in triplicate. No significant growth occurred during the course of the experiment.

### Curing of [URE3] by overexpressed proteins

Overnight SD cultures of cells carrying *YDJ1* or hybrid alleles on pRU4 for galactose induction were used to inoculate SGal medium to OD_600_ = 0.05 and incubated with shaking at 30°C. Generations were monitored as doublings of OD_600_. Cultures were sub-cultured as necessary to keep the OD_600_ less than 2.0. After 3, 6 and 9 generations of growth in galactose, aliquots were removed and cells plated onto 1/2YPD plates. Loss of [URE3] was assessed by determining the fraction of entirely red (i.e. [ure-o]) colonies after 3 days of incubation at 30°C. To test the ability of overexpressed Sis1 to block Ydj1- or Hsp104- mediated curing of [URE3], strain 1075 was co-transformed with various combinations of CEN plasmids expressing Sis1, Ydj1 or Hsp104 under the control of the GPD promoter.

### Western blotting

Cell lysates for western blots were prepared as described [77]. Briefly, cells were suspended in lysis buffer and broken by agitation with glass beads. For each sample 10 µg of protein was separated on 4–20% SDS-PAGE gels, transferred to PVDF membranes and probed using anti-c-myc antibody (AbCam #ab9106) and chemiluminescence. After developing, the blots were stained by amido-black (Sigma #A-8181) as a loading and transfer control.

### Proteins

Hsp104 [78], Ydj1 [79], and GFP-38 [80] were purified as described. SSY was isolated as described for Ydj1 [79]. Sis1 was purified as described [81] with some modifications. Briefly, BL21 (DE3) was transformed with a pET11 plasmid containing the Sis1 gene, cultures were grown at 30°C to OD_595_ 0.8 and cells were induced with 1 mM IPTG for 3 h. Clarified cell lysates were applied to an S-Sepharose-FF column (GE Healthcare) in 20 mM MES buffer, pH 6.0, 0.1 mM EDTA and 1 mM DTT. Sis1 was eluted with a linear gradient from 0–1 M NaCl. Peak fractions were pooled, buffer exchanged into 20 mM MES buffer pH 6.0, 0.1 mM EDTA and 1 mM DTT. The sample was applied to a monoS column (GE Healthcare) and eluted with a linear gradient from 0–1 M NaCl. YYS was purified similarly to Sis1, except that the buffer used was 25 mM HEPES, pH 7.6, 0.1 mM EDTA and 1 mM DTT. For Ssa1, a pET24 plasmid containing the Ssa1 gene was transformed into Rosetta BL21 (DE3) cells. Cultures were grown to 0.8 OD_595_ at 30°C and induced with 0.2 mM IPTG overnight. The clarified lysate was applied to a Q-sepharose FF column (GE Healthcare) in 20 mM Tris^.^HCl, pH 7.6, 40 mM KCl, 0.1 mM EDTA and 1 mM DTT. Ssa1was eluted with a linear gradient of 40–400 mM KCl over 20 column volumes. Peak fractions were collected and buffer exchanged into 25 mM HEPES, pH 7.6, 100 mM KCl, 0.1 mM EDTA and 1 mM DTT and further purified over a monoQ column (GE Healthcare) using a linear gradient of 100–400 mM KCl over 20 column volumes. Peak fractions were collected, analyzed, supplemented with 10% glycerol, frozen on dry ice and stored at −80°C.

#### GFP-38 reactivation

Experiments were performed as described previously [80] with some modifications. Reactivation was carried out in HKE Buffer (25 mM HEPES, 75 mM KCl, 0.1 mM EDTA, pH 7.6) and GFP-38 heat-denaturation was carried out in Buffer D (20 mM Tris·HCl, 100 mM KCl, 0.1 mM EDTA, 10% glycerol, pH 7.5). 14 mM GFP-38 in buffer D was heated at 80°C for 15 min, frozen on dry ice, thawed and immediately used in experiments. Reactivation reactions (100 µl) contained HKE buffer, 4 mM ATP, 10 mM MgCl_2_, an ATP regenerating system (10 mM creatinine phosphate and 0.03 mg/mL creatinine kinase), 0.7 µM denatured GFP-38, 0.2 µM Hsp104, 1 µM Ssa1 and 0.2 µM Hsp40 (Sis1, Ydj1, SSY or YYS). Reactions were initiated by adding MgATP, and reactivation was monitored by measuring GFP fluorescence at 23°C using a TECAN Infinite M200Pro plate reader. GFP-38 reactivation was calculated as a percentage of the initial native GFP-38 fluorescence.

#### Luciferase reactivation

Luciferase (80 nM; 55 µl) was heat-denatured in HKE buffer containing 0.05 mg/mL BSA, 2 mM DTT and 10 mM MgCl_2_ at 45°C for 7 min and then cooled to 4°C for 1 min. Denatured luciferase was added to reactivation reactions (70 µl total) containing HKE, 0.1 mg/mL BSA, 2 mM DTT, 10 mM MgCl_2_, 3 mM ATP, an ATP regenerating system as above, 2 µM Ssa1 and 1 µM Sis1, Ydj1, SSY or YYS and incubated at 23°C. Luciferase reactivation was monitored by removing 5 µL aliquots at 5 min intervals and measuring luminescence in a TECAN Infinite M200Pro plate reader in the presence of luciferin. Reactivation was determined compared to an unheated luciferase control.

## Supporting Information

Figure S1 Confirmation of presence of [*PSI^+^*] in strains shown in Fig. 1B. (A) Cells taken from FOA plates containing limiting adenine were crossed with [*psi^−^*] wild type mating tester strain 621 (*MAT alpha SUQ5 kar1-1 ade2-1 ura2*). If the prion is present (even if weak) in the BK*E strain, then it will be propagated more normally in the diploid, which expresses Hsp104. The mating plate was replica-plated onto medium selecting for diploids and containing either limiting adenine (left panel) or no adenine (right panel). The plate on the left containing adenine is a mating control that allows growth of all diploids. The plate on the right lacking adenine allows growth only of diploids that are [*PSI^+^*]. (B) Adenine phenotype of diploids is curable by growth on guanidine. Diploids from panel A were streaked onto 1/2YPD lacking (left) or containing (right) 3 mM guanidine-hydrochloride, which cures cells of prions by inactivating Hsp104 [70]. Red color of cells on the plate containing guanidine indicates loss of [*PSI^+^*]. (C) As in panel (A) except using cells shown in Figure 1C. (D) As in panel (B) except using diploids shown in panel (C).
(TIF)

Figure S2 Western blot analysis to compare abundance of wild type and hybrid proteins expressed in different strains from various promoters. All proteins contain N-terminal, c-myc epitope tags and were detected by probing with anti-myc antibodies. (A) Proteins indicated at top expressed from the GPD (*TDH3*) promoter on single-copy plasmids. (B) Proteins expressed from the *GAL1* promoter from cells grown in galactose for 6 hours. (C) Proteins expressed from the *SIS1* promoter on single-copy plasmids (left of vertical line) or from the GPD promoter on high-copy plasmids (right of vertical line). ev, empty vector; blank, no sample in lane.
(TIF)

Figure S3 Plasmid shuffle using more abundantly expressed proteins. Cells expressing proteins from *SIS1* or GPD promoter (as indicated) were replica-plated onto FOA and incubated at the indicated temperatures. Each patch of cells is from a separate transformant colony. Cells expressing wild type Sis1 from the GPD promoter were recovered on FOA plates even at 34°C. In contrast, the hybrid proteins containing the CTD of Sis1 not only failed to complement the growth defect, but also caused cells to grow more slowly than those with the empty vector on the FOA plates at 25°C. Thus, increasing expression of these hybrids inhibited growth. Abundance of the Sis1 CTD-containing hybrid proteins in transformants expressing proteins from the GPD promoter on high-copy plasmids was comparable to those containing the Ydj1 CTD (Figure S2C, compare right and left sets of lanes). Although transformants expressing these proteins were obtained readily in *ydj1Δ* cells with the plasmid encoding wild type Ydj1 (e.g. used as source of cells for the blot), none of them could be recovered on FOA at any temperature. These results indicate that cells expressing high levels of these proteins depended on Ydj1 to remain viable. Thus, these full-length proteins caused a dose-dependent inhibition of growth in cells lacking Ydj1.
(TIF)

Figure S4 *E. coli* disaggregation machinery BKE cannot propagate [URE3]. [*PSI^+^*] strain 1408, which expresses Hsp104 from a URA3-based plasmid to propagate [*PSI^+^*] and various combinations of empty vectors (ev), ClpB (B), DnaK (K) and GrpE (E). Transformants were grown on medium containing uracil to allow loss of Hsp104 and then replica-plated onto FOA plates (shown) containing limiting adenine, which allows growth of all cells without the *URA3* plasmid, and lacking adenine, which allows growth only of ura^−^ cells propagating the prion. Lower panels show a similar experiment using transformants of strain 1410, which initially propagated [URE3]. The combination of BKE propagates [*PSI^+^*], but not [URE3].
(TIF)
