# Supplementary figures and images for: Hsp40s Specify Functions of Hsp104 and Hsp90 Protein Chaperone Machines
Source: PLoS Genet. 2014 Oct 16;10(10):e1004720. doi: 10.1371/journal.pgen.1004720 (PMC4199505; doi:10.1371/journal.pgen.1004720)

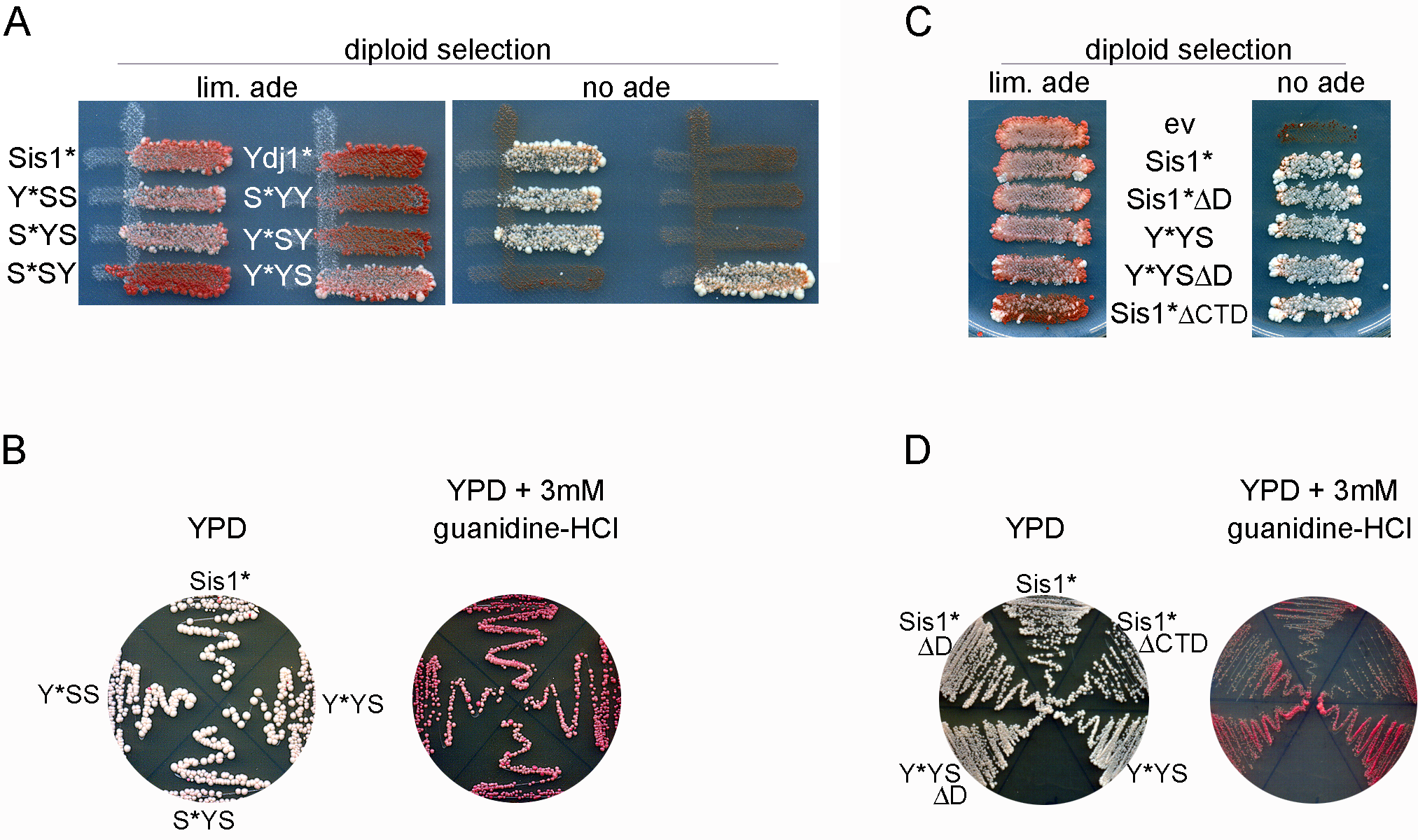

Supplement: Figure S1 — Confirmation of presence of [PSI+] in strains shown in Fig. 1B. (A) Cells taken from FOA plates containing limiting adenine were crossed with [psi−] wild type mating tester strain 621 (MAT alpha SUQ5 kar1-1 ade2-1 ura2). If the prion is present (even if weak) in the BK*E strain, then it will be propagated more normally in the diploid, which expresses Hsp104. The mating plate was replica-plated onto medium selecting for diploids and containing either limiting adenine (left panel) or no adenine (right panel). The plate on the left containing adenine is a mating control that allows growth of all diploids. The plate on the right lacking adenine allows growth only of diploids that are [PSI+]. (B) Adenine phenotype of diploids is curable by growth on guanidine. Diploids from panel A were streaked onto 1/2YPD lacking (left) or containing (right) 3 mM guanidine-hydrochloride, which cures cells of prions by inactivating Hsp104 [70]. Red color of cells on the plate containing guanidine indicates loss of [PSI+]. (C) As in panel (A) except using cells shown in Figure 1C. (D) As in panel (B) except using diploids shown in panel (C). (TIF) [file pgen.1004720.s001.tif]

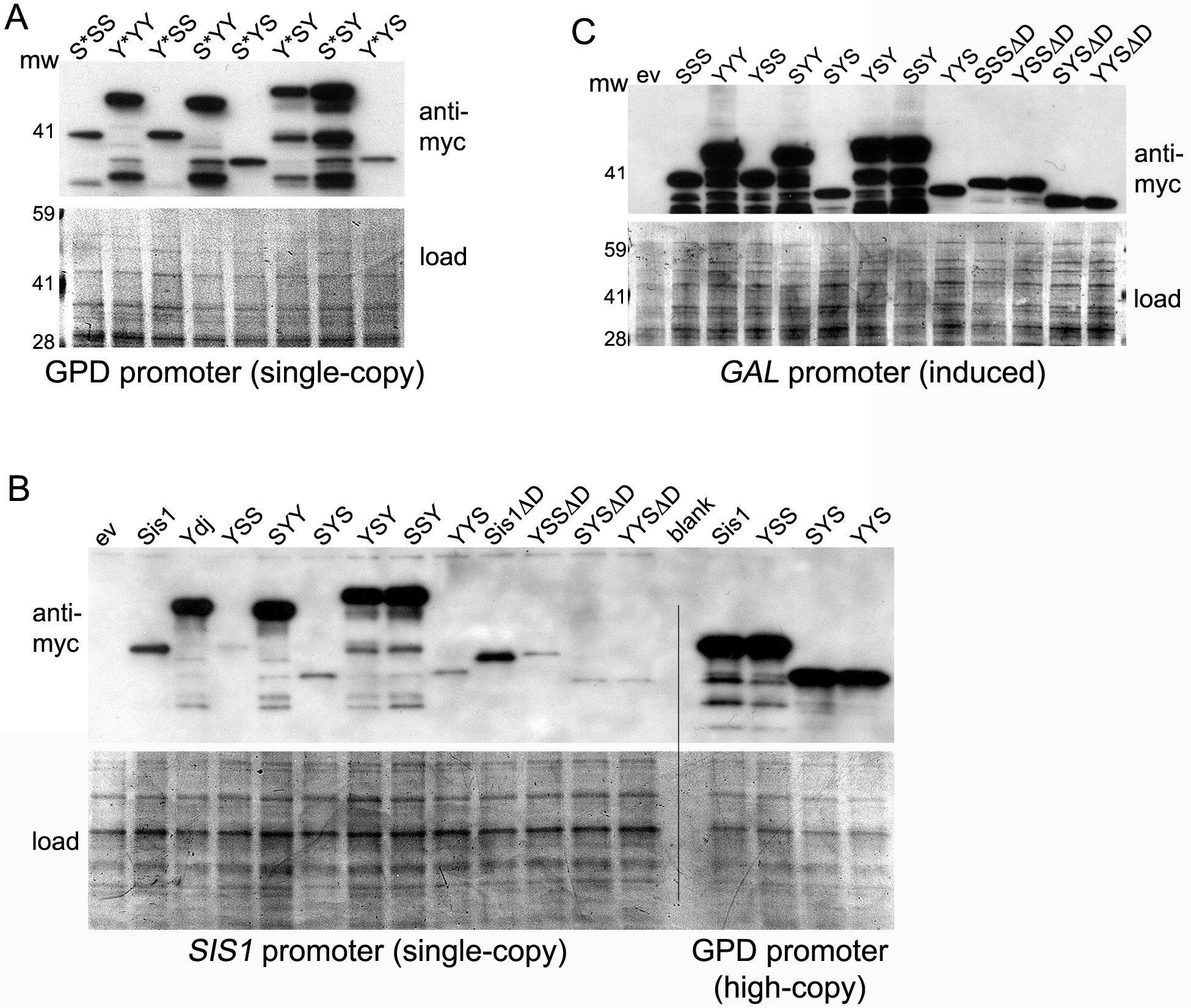

Supplement: Figure S2 — Western blot analysis to compare abundance of wild type and hybrid proteins expressed in different strains from various promoters. All proteins contain N-terminal, c-myc epitope tags and were detected by probing with anti-myc antibodies. (A) Proteins indicated at top expressed from the GPD (TDH3) promoter on single-copy plasmids. (B) Proteins expressed from the GAL1 promoter from cells grown in galactose for 6 hours. (C) Proteins expressed from the SIS1 promoter on single-copy plasmids (left of vertical line) or from the GPD promoter on high-copy plasmids (right of vertical line). ev, empty vector; blank, no sample in lane. (TIF) [file pgen.1004720.s002.tif]

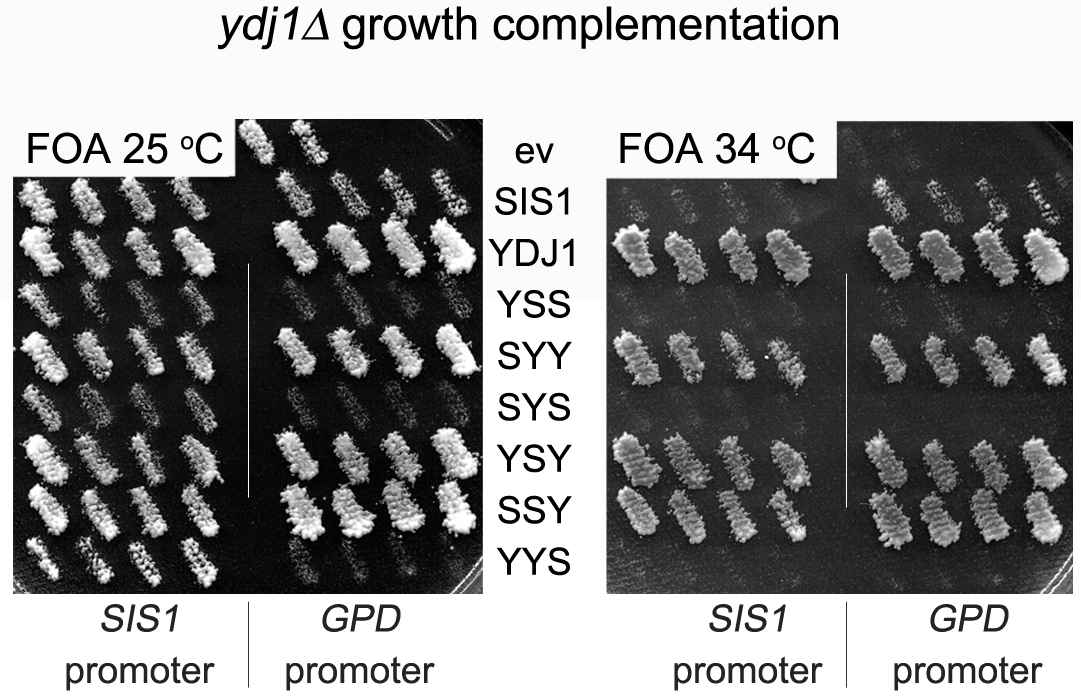

Supplement: Figure S3 — Plasmid shuffle using more abundantly expressed proteins. Cells expressing proteins from SIS1 or GPD promoter (as indicated) were replica-plated onto FOA and incubated at the indicated temperatures. Each patch of cells is from a separate transformant colony. Cells expressing wild type Sis1 from the GPD promoter were recovered on FOA plates even at 34°C. In contrast, the hybrid proteins containing the CTD of Sis1 not only failed to complement the growth defect, but also caused cells to grow more slowly than those with the empty vector on the FOA plates at 25°C. Thus, increasing expression of these hybrids inhibited growth. Abundance of the Sis1 CTD-containing hybrid proteins in transformants expressing proteins from the GPD promoter on high-copy plasmids was comparable to those containing the Ydj1 CTD (Figure S2C, compare right and left sets of lanes). Although transformants expressing these proteins were obtained readily in ydj1Δ cells with the plasmid encoding wild type Ydj1 (e.g. used as source of cells for the blot), none of them could be recovered on FOA at any temperature. These results indicate that cells expressing high levels of these proteins depended on Ydj1 to remain viable. Thus, these full-length proteins caused a dose-dependent inhibition of growth in cells lacking Ydj1. (TIF) [file pgen.1004720.s003.tif]

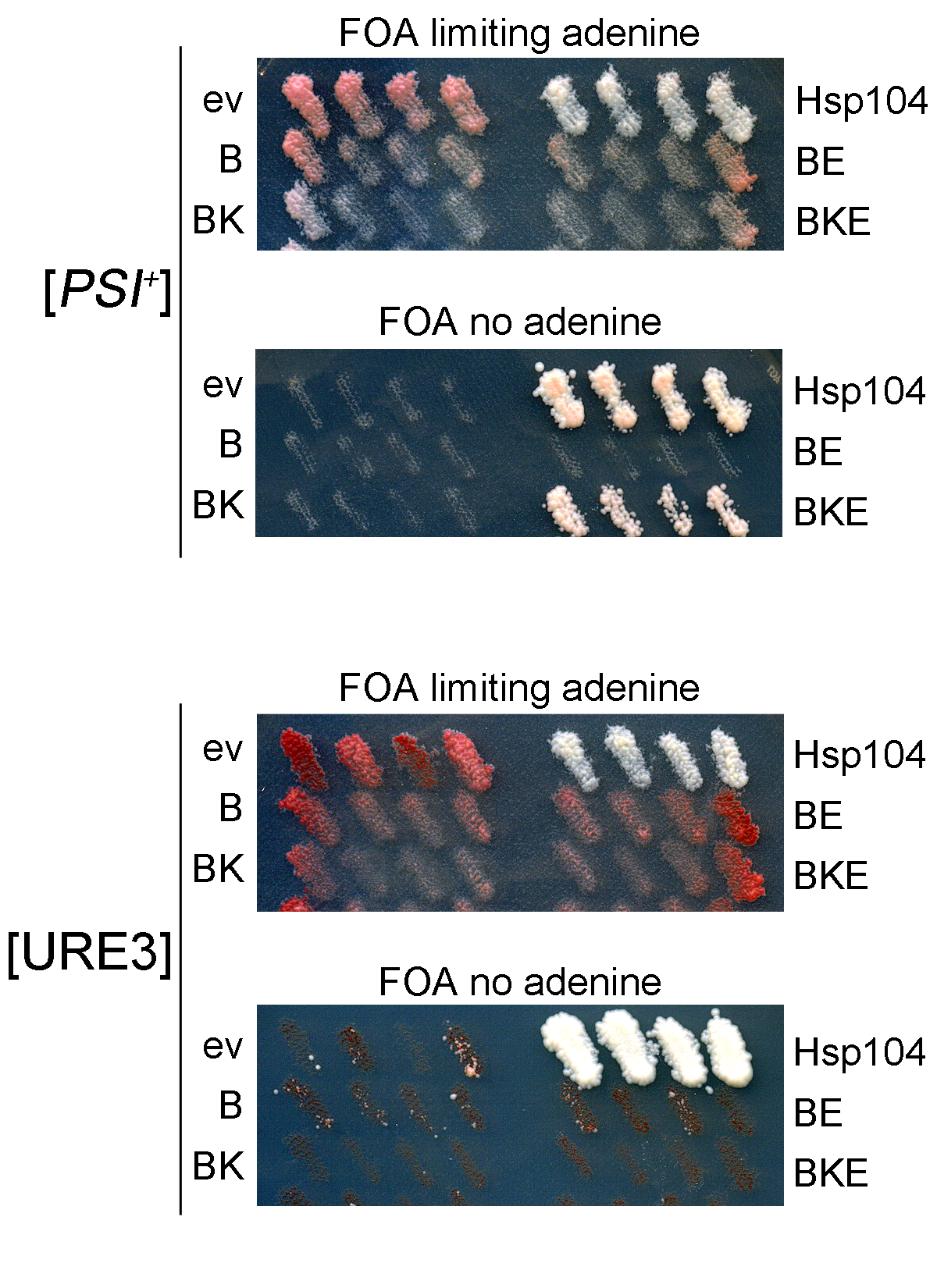

Supplement: Figure S4 — E. coli disaggregation machinery BKE cannot propagate [URE3]. [PSI+] strain 1408, which expresses Hsp104 from a URA3-based plasmid to propagate [PSI+] and various combinations of empty vectors (ev), ClpB (B), DnaK (K) and GrpE (E). Transformants were grown on medium containing uracil to allow loss of Hsp104 and then replica-plated onto FOA plates (shown) containing limiting adenine, which allows growth of all cells without the URA3 plasmid, and lacking adenine, which allows growth only of ura− cells propagating the prion. Lower panels show a similar experiment using transformants of strain 1410, which initially propagated [URE3]. The combination of BKE propagates [PSI+], but not [URE3]. (TIF) [file pgen.1004720.s004.tif]
